# Shaping the evolutionary tree of green plants: evidence from the GST family

**DOI:** 10.1038/s41598-017-14316-w

**Published:** 2017-10-30

**Authors:** Francesco Monticolo, Chiara Colantuono, Maria Luisa Chiusano

**Affiliations:** 1 Department of Agricultural Sciences, University of Naples “Federico II”, 80055 Portici (Na), Italy; 2 Research Infrastructures for Marine Biological Resources (RIMAR), Stazione Zoologica Anton Dohrn, Villa Comunale, 80121 Naples, Italy

## Abstract

Glutathione-S-transferases (GSTs) are encoded by genes belonging to a wide ubiquitous family in aerobic species and catalyze the conjugation of electrophilic substrates to glutathione (GSH). GSTs are divided in different classes, both in plants and animals. In plants, GSTs function in several pathways, including those related to secondary metabolites biosynthesis, hormone homeostasis, defense from pathogens and allow the prevention and detoxification of damage from heavy metals and herbicides. 1107 GST protein sequences from 20 different plant species with sequenced genomes were analyzed. Our analysis assigns 666 unclassified GSTs proteins to specific classes, remarking the wide heterogeneity of this gene family. Moreover, we highlighted the presence of further subclasses within each class. Regarding the class GST-Tau, one possible subclass appears to be present in all the Tau members of ancestor plant species. Moreover, the results highlight the presence of members of the Tau class in *Marchantiophytes* and confirm previous observations on the absence of GST-Tau in *Bryophytes* and green algae. These results support the hypothesis regarding the paraphyletic origin of *Bryophytes*, but also suggest that *Marchantiophytes* may be on the same branch leading to superior plants, depicting an alternative model for green plants evolution.

## Introduction

Glutathione-*S*-transferases (GSTs) are enzymes encoded by a ubiquitous gene family in aerobic species, able to conjugate electrophilic xenobiotics and endogenous cell components with glutathione (GSH)^1^. GSTs in plants are composed of two subunits with a molecular mass of around 25–29 kD^2^.

Initially, plant GSTs were identified in *Zea mays* for their involvement in defense mechanisms against damage by herbicide^3^. The importance of GSTs in herbicide tolerance has been demonstrated expressing maize GSTs in tobacco plants. The treated plants were revealed to have a greater herbicide tolerance compared to untreated tobacco plants^4^. GSTs can also act as detoxifying agents from endogenous cell components. For example, *Bronze 2* in maize has been demonstrated to be involved in anthocyanin transport into cytoplasmic vacuoles^5^. A similar behavior has been highlighted for *An9* in *Petunia hybrida* ^6^, *TT19* in *Arabidopsis thaliana* ^7^, *PGSC0003DMG400016722* in *Solanum tuberosum* ^8^ and *DQ198153* in *Citrus sinensis*, cultivar Moro nucellare^9^, suggesting that, probably, GSTs act in the last step of the anthocyanin biosynthetic pathway^10^, when these molecules are transported to the vacuole.

GSTs are also important for the prevention of heavy metals damage, facilitating their storage in the vacuole. In particular, a truncated isoform of the protein encoded by *Bronze 2* in maize has a high affinity for heavy metals^11^. Moreover, GSTs may take part in the hydrogen peroxide detoxification^12^.

GSTs have a high affinity for auxins and cytokinins and this suggests that GSTs are important for hormone homeostasis and in plant defense against pathogens^2,13^. In fact, in *Solanum tuberosum*, the plants infected with the pathogen fungus *Phytophthora infestans* revealed a fast increase in the *prp 1-1* GST content, accompanied by the increase of intracellular auxin levels, suggesting the association of the phenomena to infection defense^13^.

Initially, plant GSTs were classified into four categories, type I, II, III and IV, based on amino acids sequence identity and on the conservation of the gene structure^14,15^. This classification was modified into 7 GST classes: 6 cytoplasmic classes (Tau, Phi, Zeta, Theta, Lambda and Dhar) and a further microsomal class (Mapeg)^2,16^.

Tau and Phi classes are considered plant specific classes, being the most representative in terms of the number of sequences^16^. In 2016, Munyampundu *et al*. demonstrated that the Phi class is also present in bacteria, fungi and protists. Tau and Phi classes link a wide range of xenobiotics^16^, or endogenous cell components^17^. These components function as glutathione peroxidases (GPOXs), as flavonoid-binding proteins^6–9^, and as stress-signaling proteins^18^. Moreover, the Tau class expansion appears to be associated with plant adaptation to land living^19^.

The Zeta class is linked to tyrosine degradation, catalyzing the GSH-dependent conversion of malelyacetoacetate to fumarylacetoacetate. The Theta class is similar to the corresponding mammalian class^9^ and it is present in bacteria, insects, plants, fish, and mammals^20^.

Lambda and Dhar classes were identified comparing the human Omega GSTs versus the Arabidopsis genome^17^.

Finally, the Mapeg class includes the microsomal GSTs, with transferase and peroxidase activities^21^.

Recently more 6 GST classes have been identified in plants: TCHQD, EF1Bγ, URE2p, Omega-like, Iota and Hemerythrin^19^. Members of the URE2p class were found in *Physcomitrella patens*, in *Selaginella moellendorffii* and in bacteria, probably because of horizontal gene transfer events in bacteria, while the Iota GST class was found only in *Physcomitrella patens* and in *Selaginella moellendorffii* ^19^. Hemerythrin GSTs are non-heme iron binding proteins found in metazoans, prokaryotes, protozoans, and fungi^22^, which acts in detoxification from heavy metals by catalyzing the conjugation of GSH with metal ions^19^.

A phylogenetic analysis made both in monocots (maize and rice) and in dicots (soya and Arabidopsis) demonstrated that Zeta and Theta classes are monophyletic groups in monocots, dicots and mammals, suggesting that their origin might be anterior to the division between plants and animals^23^. Zeta and Theta classes have undergone one or two duplication events, presenting at maximum three paralogs in maize, rice, soya and Arabidopsis. Phi and Tau classes show differences between monocots and dicots due to the extensive gene duplication events that monocots and dicots underwent after their divergence. Extensive duplications also resulted in genic clusters sharing high similarity in small genome regions. The reasons of these retained extensive gene duplications are still unknown^23^.

1107 GSTs from 20 different plant species with sequenced genomes were analyzed (Table 1) to reveal the organization of this relevant family in plants. Two green algae genomes, two *Bryophytes*, one *Marchantiophyta*, one *Lycopodiophyta*, one *Gymnosperm*, three monocots, ten dicots, including the reference plant species *Arabidopsis thaliana* (family *Brassicaceae*), were examined.

**Table 1:** List of plants considered for this study. Scientific name (name) of the organisms considered, their classification (A (CHL): Algae Chlorophyta, A (CHA): Algae Charophyta, B: Bryophyta, L: Lycophyta, MA: Marchantiophyta, G: Gymnosperms, M: Monocots, D: Dicots), number of chromosomes (Chr), genome size estimation in Mb (Genome), total number of genes currently estimated (Gene), genomics resource, bibliographical reference (Source + Reference) and publication year (Year).

Name	Type	Chr (n)	Genome (Mb)	Gene (n)	Source + Reference	Year
*Vitis vinifera*	D	19	475	30434	Cribi (v2) Jaillon *et al*.	2007
*Solanum tuberosum*	D	12	844	39031	Spud db (PGSC_DM_v_3.4) The Potato Genome Sequencing Consortium	2011
*Solanum lycopersicum*	D	12	900	34727	SGN (iTAG2.4) The Tomato Genome Consortium	2012
*Populus trichocarpa*	D	19	422.9	45778	Phytozome 11 (v3.0) Tuskan *et al*.	2006
*Glycine max*	D	20	1115	46430	Gramene Schmuz *et al*.	2010
*Coffee canephora*	D	11	710	25574	Coffee genome Hub Denoeud *et al*.	2014
*Citrus sinensis*	D	9	367	29445	Licciardello *et al*. Xu *et al*.	2012
*Capsicum annum*	D	12	3349	35336	SGN (v1.55) Qin *et al*.	2014
*Arabidopsis thaliana*	D	5	125	25498	TAIR10 The Arabidopsis Genome Initiative	2000
*Amborella trichopoda*	D	13	870	14000	Phytozome 11 (v1.0) Amborella Genome Project	2013
*Zea mays*	M	10	2300	32540	Phytozome 11 (Ensembl-18) Schnable *et al*.	2009
*Spirodela polyrhiza*	M	20	158	19623	Phytozome 11 (v2) Wang *et al*.	2013
*Oryza sativa*	M	12	420	29961	TIGR Goff *et al*.	2005
*Picea abies*	G	12	19600	28354	Congenie (v1) Nystedt *et al*.	2013
*Selaginella moellendorffii*	L	27	212.5	22285	Phytozome 11 (v1.0)	
Banks *et al*.	2011					
*Marchantia polymorpha*	MA	/	225.8	19287	Phytozome 11 (v3.1) https://phytozome.jgi.doe.gov	2016
*Sphagnum fallax*	B	/	395	26939	Phytozome 11 (v0.5) https://phytozome.jgi.doe.gov	2015
*Physcomitrella patens*	B	27	510	35938	Liu *et al*. Rensing *et al*.	2008
*Klebsormidium flaccidum*	A (CHA)	22–26	117.1 ± 21.8	16215	CGA Hori *et al*.	2014
*Micromonas pusilla CCMP1545*	A (CHL)	17	21.95	10575	Phytozome 11 (v3.0) Worden *et al*.	2009

## Results

### Class assignment of unclassified GSTs

The collection of 1107 GST protein sequences from the 20 species consisted of 214 Tau, 53 Phi, 41 Theta, 7 Lambda, 23 Dhar, 28 Zeta, 21 Mapeg, 10 Hemerythrin, 15 EF-gamma, 4 URE2p, 9 TCHQD, 2 Iota and 16 Omega-like GSTs. In addition, 666 unclassified GSTs were also included (Table 2, numbers in brackets).

**Table 2:** Number of GSTs per species and per class. Type classes as in Table 1.

	Type	Tot	TAU	PHI	THETA	LAMBDA	DHAR	ZETA	MAPEG	HEMERY-THRIN	El-F2 gamma	URE2p	TCHQD	IOTA	Omega-like	Not classified before the analysis
*Vitis vinifera*	D	132	88 (96)	13 (11)	2 (2)	2 (/)	2 (3)	16 (10)	3 (/)	/ (/)	2 (/)	/ (/)	1 (1)	/ (/)	3 (/)	9
*Solanum tuberosum*	D	88	58 (/)	5 (/)	3 (/)	6 (1)	2 (5)	8 (2)	1 (1)	/ (/)	1 (/)	/ (/)	1 (1)	/ (/)	2 (/)	78
*Solanum lycopersicum*	D	86	68 (4)	5 (1)	/ (10)	2 (/)	3 (/)	3 (/)	1 (1)	/ (/)	1 (/)	/ (/)	1 (1)	/ (/)	2 (/)	69
*Populus trichocarpa*	D	79	66 (/)	6 (/)	2 (/)	1 (/)	/ (/)	1 (1)	1 (2)	/ (/)	2 (2)	/ (/)	/ (/)	/ (/)	/ (/)	74
*Glycine max*	D	15	12 (12)	1 (1)	/ (/)	/ (/)	/ (/)	2 (2)	/ (/)	/ (/)	/ (/)	/ (/)	/ (/)	/ (/)	/ (/)	/
*Coffee canephora*	D	54	34 (12)	3 (2)	7 (7)	/ (/)	2 (2)	4 (4)	2 (2)	/ (/)	1 (/)	/ (/)	1 (1)	/ (/)	/ (/)	34
*Citrus sinensis*	D	25	12 (12)	10 (10)	/ (/)	1 (1)	/ (/)	1 (1)	1 (1)	/ (/)	/ (/)	/ (/)	/ (/)	/ (/)	/ (/)	/
*Capsicum annum*	D	39	30 (3)	4 (/)	1 (5)	2 (1)	/ (/)	1 (/)	1 (1)	/ (/)	/ (/)	/ (/)	/ (1)	/ (/)	/ (/)	28
*Arabidopsis thaliana*	D	70	28 (28)	15 (15)	3 (3)	3 (3)	3 (3)	4 (4)	3 (3)	/ (/)	/ (/)	/ (/)	2 (2)	/ (/)	9 (9)	/
*Amborella trichopoda*	D	52	36 (/)	5 (/)	1 (1)	3 (/)	1 (1)	2 (/)	/ (/)	/ (/)	2 (2)	/ (/)	/ (/)	/ (/)	2 (/)	48
*Zea mays*	M	55	30 (1)	7 (1)	1 (1)	/ (/)	4 (3)	5 (/)	1 (1)	/ (/)	2 (2)	/ (/)	/ (/)	/ (/)	5 (/)	46
*Spirodela polyrhiza*	M	29	11 (/)	6 (/)	1 (/)	/ (/)	2 (/)	4 (1)	1 (1)	/ (/)	1 (1)	/ (/)	1 (/)	/ (/)	2 (/)	26
*Oryza sativa*	M	80	52 (5)	18 (1)	1 (/)	/ (/)	2 (/)	5 (/)	1 (1)	/ (/)	/ (/)	/ (/)	1 (/)	/ (/)	/ (/)	73
*Picea abies*	G	104	73 (/)	9 (/)	1 (/)	4 (/)	2 (/)	9 (/)	/ (/)	1 (/)	4 (/)	/ (/)	1 (/)	/ (/)	1 (/)	104
*Selaginella moellendorffii*	L	60	39 (40)	1 (1)	3 (3)	/ (/)	3 (2)	1 (1)	2 (2)	1 (1)	1 (1)	3 (3)	/ (/)	1 (1)	5 (5)	/
*Marchantia polymorpha*	MA	34	2 (1)	15 (/)	3 (/)	/ (/)	1 (/)	3 (/)	2 (1)	1 (1)	1 (1)	1 (/)	2 (1)	1 (/)	2 (1)	28
*Sphagnum fallax*	B	38	/	1 (/)	6 (6)	7 (/)	1 (1)	2 (1)	4 (3)	5 (/)	2 (1)	7 (/)	/ (/)	/ (/)	3 (/)	26
*Physcomitrella patens*	B	37	/	10 (10)	3 (3)	1 (1)	3 (3)	1 (1)	/ (/)	8 (8)	4 (4)	1 (1)	5 (5)	1 (1)	/ (/)	/
*Klebsormidium flaccidum*	A (CHA)	16	1 (/)	3 (/)	5 (/)	/ (/)	/ (/)	1 (/)	1 (/)	/ (/)	/ (/)	2 (/)	1 (/)	1 (/)	1 (/)	16
*Micromonas pusilla CCMP1545*	A (CHL)	14	2 (/)	1 (/)	/ (/)	1 (/)	/ (/)	4 (/)	/ (1)	/ (/)	2 (1)	/ (/)	/ (/)	2 (/)	2 (1)	10
Total		1107	643	138	43	33	31	77	25	16	26	14	17	6	39	666

In brackets the number of GSTs per class before the assignment resulting from the reported analyses.

**Figure 1:**
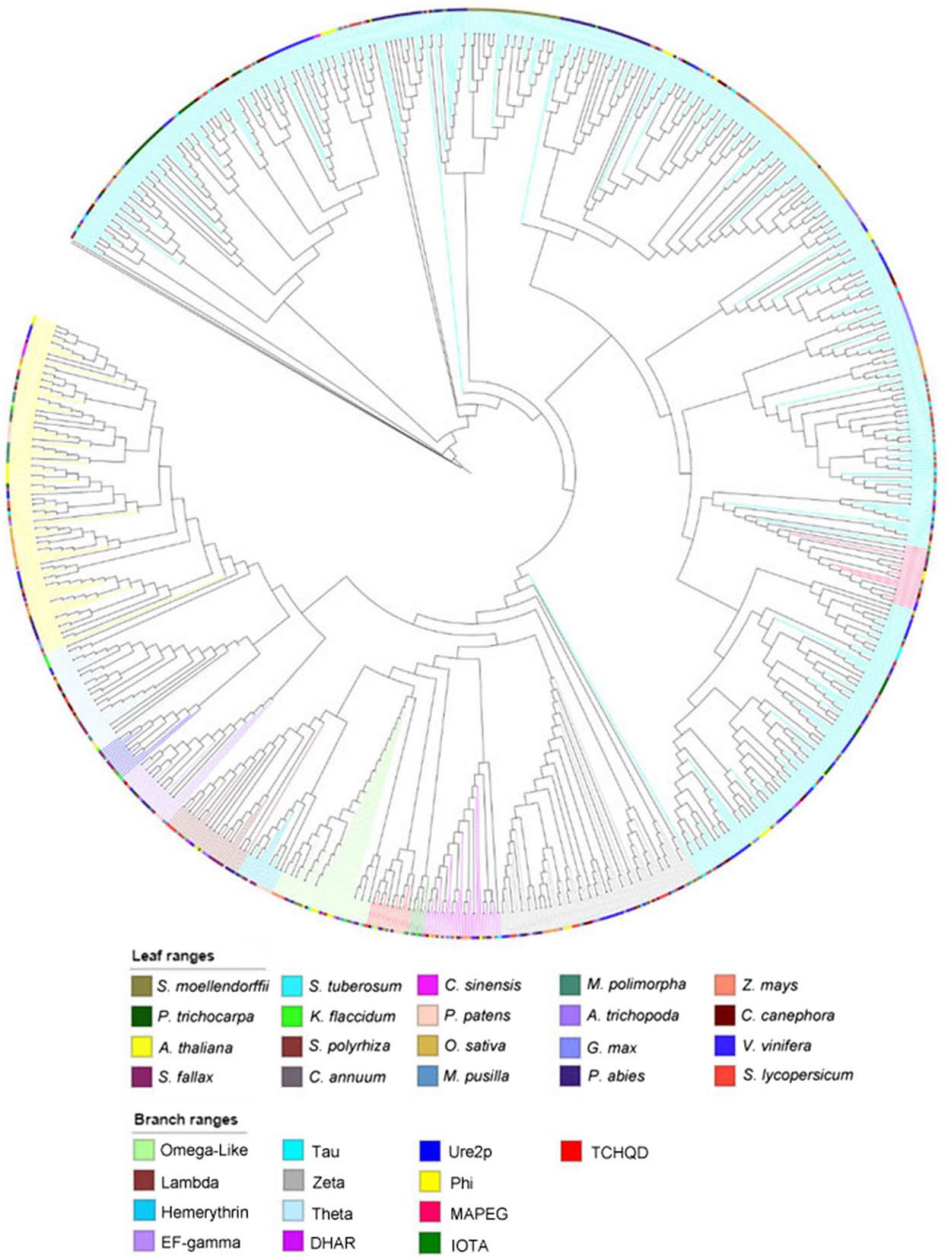
Phylogenetic tree of all the 1107 GSTs. Colors of the leaves indicate the species, while those of the branches indicate the GST class, as reported in the corresponding legends.

In order to associate the unclassified GSTs with specific classes, the collection was analyzed by a multiple protein sequence alignment using Muscle^24^ and an associated phylogenetic tree based on the maximum likelihood method^25^ (Fig. 1). The analysis defined the class association of the 666 unclassified GSTs (Table 2, numbers non in brackets), highlighting the presence of GST-Tau in *Chlorophytes, Marchantiophytes* and in *Klebsormidiales*, and confirming results from Liu *et al*., 2013, concerning their absence in *Bryophytes*.

### Plant phylogeny depicted by GSTs

It can be noted (Fig. 1) that one GST (kfl00659_0030) from *Klebsormidium flaccidum* (*Klebsormidiales*) and two GSTs (213211, 49816) from *Micromonas pusilla* (*Chlorophyta*) resulted in the Tau class, as also summarized in Table 2.

In Liu *et al*., 2013, the authors suggested that GST-Tau genes were absent in algae and *Bryophytes* and served in *Tracheophytes* to colonize lands. Interestingly, our preliminary results show also that two GSTs (Mapoly0031s0032.1, Mapoly0118s0009.1) of *Marchantia polymorpha* (*Marchantiophyta*) belong to the Tau class.

In Table 3 the results of further analyses on the assignment of these 5 sequences to a specific GST class are shown. A BLASTp analysis^26^, versus all the other GST protein sequences here collected and versus the UNIPROTkb^27^ database, highlighted that the two *Marchantia polymorpha* (Mapoly0031s0032.1, Mapoly0118s0009.1) GST-Tau sequences are actually significantly similar to other members of the Tau class. This result is also valid for one of the two *Micromonas pusilla* (213211) sequences, although with lower significance (low score and identity values).

**Table 3:** Summary of the two BLASTp results.

GST Collection					UniProt				
Best hits	GST Class	Organism	Score	E-value	Best hits	GST Class	Organism	Score	E-value
**Mapoly0031s0032.1 M. polymorpha**					**Mapoly0031s0032.1 M. polymorpha**				
MA_944351p0010	Tau	*P.abies*	144	2.00E-44	A0A176VUP3	uncharacterized GST	*M.polymorpha*	1295	1.00E-178
MA_8564957p0010	Tau	*P.abies*	144	2.00E-44	A0A0C9RTV3	Transcribed RNA	*W.nobilis*	357	2.30E-37
MA_213889p0010	Tau	*P.abies*	138	5.00E-42	L7S1R3	Tau	*P.tabuliformis*	328	4.60E-33
**Mapoly0118s0009.1 M. polymorpha**					**Mapoly0118s0009.1 M. polymorpha**				
MA_34977p0010	Tau	*P.abies*	162	3.00E-51	A0A176WNU4	uncharacterized GST	*M.polymorpha*	1140	7.40E-155
MA_213889p0010	Tau	*P.abies*	157	3.00E-49	A0A0C9RTV3	Transcribed RNA	*W.nobilis*	414	8.30E-46
MA_160708p0010	Tau	*P.abies*	157	3.00E-49	L7S309	Tau	*P.tabuliformis*	395	6.30E-43
**kfl00659_0030 K. flaccidum**					**kfl00659_0030 K. flaccidum**				
Sphfalx0108s0054.1	MAPEG	*S.fallax*	36.6	4.00E-05	K9TE82	putative MAPEG	*O.acuminata*	203	7.20E-17
Sphfalx0011s0245.1	MAPEG	*S.fallax*	32.3	0.001	L8N7J9	MAPEG	*P.biceps*	194	1.30E-15
Sphfalx0077s0049.1	MAPEG	*S.fallax*	30.4	0.005	A0A0M1JQ19	putative MAPEG	*Planktothricoides*	185	2.50E-14
**213211 M. pusilla**					**213211 M. pusilla**				
AT1G78370.1	Tau	*A.thaliana*	79	4.00E-19	C1MVD9	putative OMEGA-like	*M.pusilla*	1582	0
AT1G78380.1	Tau	*A.thaliana*	78.2	7.00E-19	C1EG60	putative OMEGA-like	*M.commoda*	1182	5.80E-160
Cc01_g15350	Tau	*C.canephora*	78.2	8.00E-19	A4SB04	putative OMEGA-like	*O.lucimarinus*	979	3.00E-129
**49816 M. pusilla**					**49816 M. pusilla**				
PGSC0003DMP400034285	MAPEG	*S.tuberosum*	84.3	4.00E-23	C1MGH6	MAPEG	*M.pusilla*	836	7.30E-112
LOC_Os03g50130.1	MAPEG	*O.sativa*	83.2	9.00E-23	C1EIA6	putative MAPEG	*M.commoda*	373	7.20E-42
Solyc02g081430.2.1	MAPEG	*S.lycopersicum*	82.8	1.00E-22	T1P743	MAPEG	*P.minimum*	317	1.50E-33

Two sequences from *Marchantia polymorpha*, one sequence from *Klebsormidium flaccidum* and two sequences from *Micromonas pusilla* were compared versus the GST protein sequences here collected and the UniProtkb database.

On the other hand, the sequence from *Klebsormidium flaccidum* (kfl00659_0030) and the remaining one from *Micromonas pusilla* (49816) showed a significant alignment with members of the Mapeg class (Table 3).

A domain search using the Interpro tool^28^ (Figure S1) showed that a GST-Tau from both the phylogenetic tree and the BLASTp analysis in *Micromonas pusilla* (213211) is actually an Omega-like GST (Figure S1).

**Figure 2:**
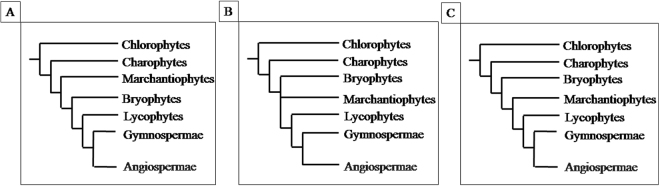
(**A**) Phylogenetic tree currently proposed for green plants evolution. (**B**) Green plants evolutionary tree resulting from Cooper 2014. (**C**) Green plants evolutionary tree proposed herein.

The presence of the GST-Tau class in plants from *Lycophytae* to higher plants in Liu *et al*., 2013, suggested that this class of proteins served the plants to colonize lands. The absence of Tau GSTs in all *Bryophytes* by a multiple sequence alignment and an associated phylogenetic tree of all the available GSTs from this division and the 1107 proteins from our collection (data not shown) was confirmed. This study highlighted the presence of two Tau GSTs in the *Marchantiophytes* division. This evidence supports the hypothesis of a paraphyletic origin for *Bryophytes* ^29–31^ (Fig. 2), in contrast with the general assumption that *Bryophytes* and *Marchantiophytes* are a separated clade from the one that gave rise to higher plants, and it also suggests that *Marchantiophytes* could indeed belong to the branching bringing to higher plants.

### Tau subclasses

**Figure 3:**
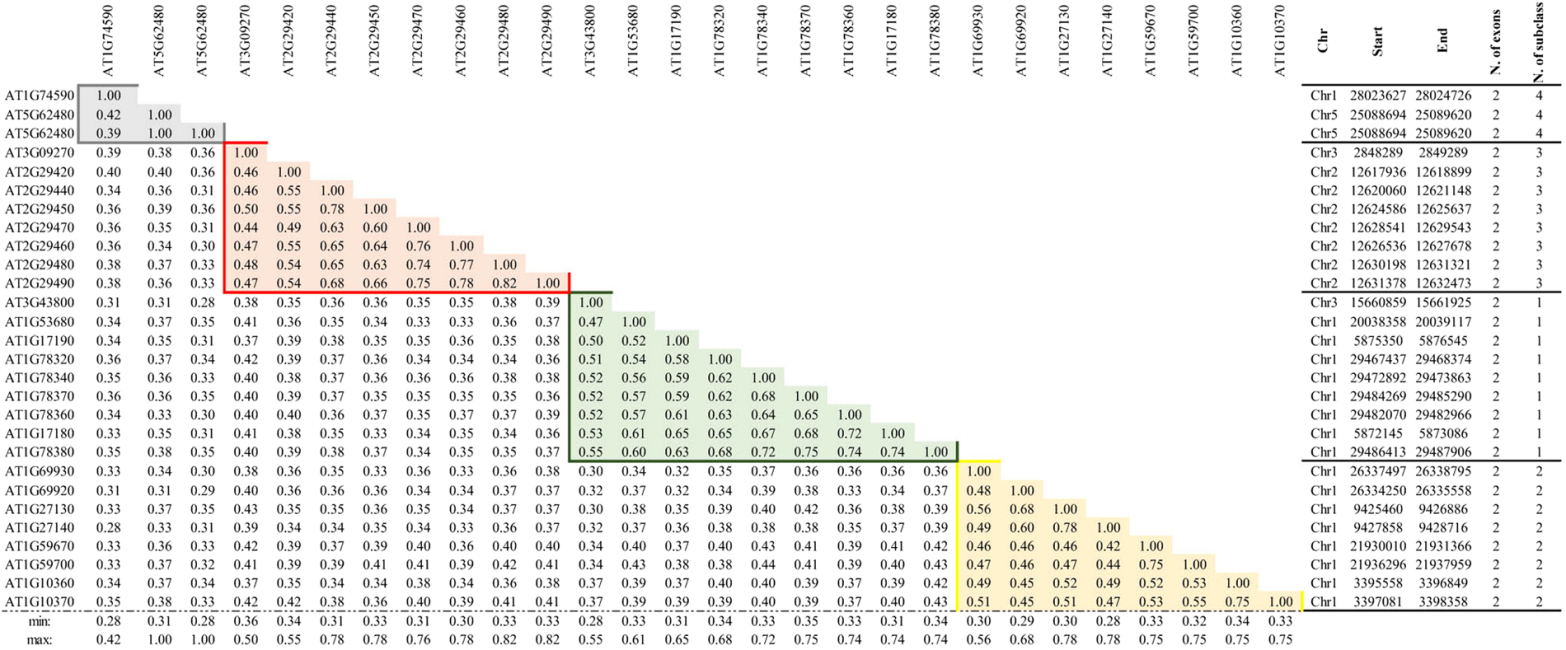
*Arabidopsis thaliana* GST-Tau similarity matrix. Minimum and maximum values per column are indicated. The last columns indicate annotation of the gene in terms of chromosome (Chr), gene start (Start) and gene end (End), number of exons per gene (N. of exons) and the assignment to the identified subclass (Subclass number).

Data collected in this research clearly highlights the amplification of the GST-Tau class when compared to other GST classes^8^ (Fig. 1). In the work of Wagner^32^, the authors suggested that GST-Tau in Arabidopsis could be divided into three subclasses. In order to further investigate the expansion of the Tau class, a pairwise similarity of these proteins in *Arabidopsis thaliana* (Fig. 3) and in *Solanum lycopersicum* (Table S2), respectively, was carried out. The results highlight the presence of four subclasses in Arabidopsis (Fig. 3), one more than what Wagner^32^ described. Whereas five subclasses were identified in tomato (Table S2).

**Figure 4:**
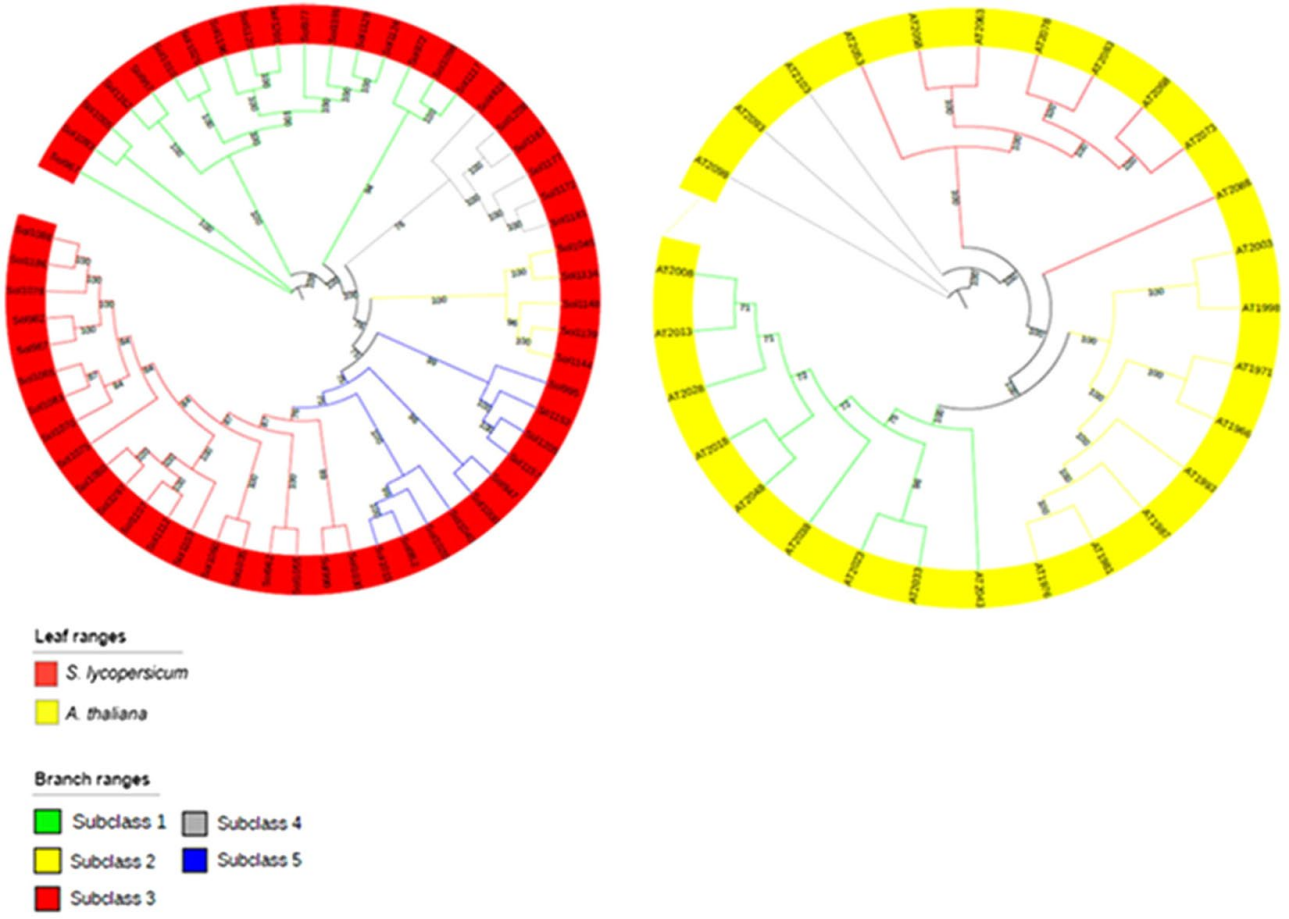
Phylogenetic tree of GSTs from the class Tau in tomato (red) and Arabidopsis (yellow). The branches indicate the possible different subclasses, according to their color reported in the legend. Bootstrap values are also indicated.

**Figure 5:**
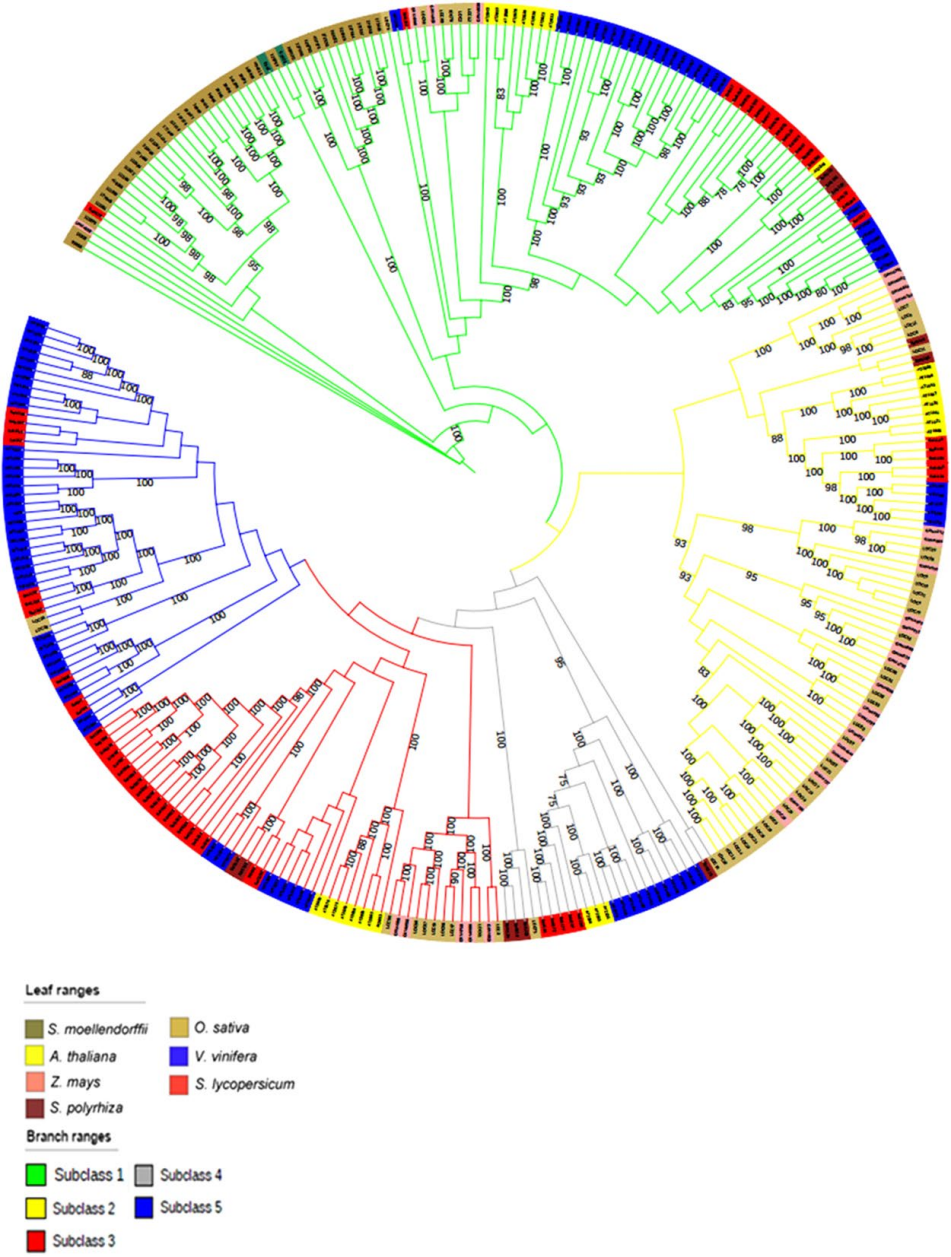
Phylogenetic tree of GSTs from class Tau of nine different species (as reported in the leaves legend). The branches indicate the possible different subclasses, according to the color reported in the corresponding legend. Bootstrap values are also indicated.

For further confirmation, two independent phylogenetic trees, one for Arabidopsis and one for tomato (Fig. 4), respectively, were drawn. The trees support our results from the pairwise similarity matrices. Successively, a phylogenetic tree (Fig. 5) with a reduced number of species, when compared to the one in Fig. 1, and including only Arabidopsis, *S. lycopersicum, V. vinifera*, three monocots (maize, rice and greater duckweed), *S. moellendorffii* and *M. polymorpha* was built. The latter two species are considered plants ancestors^33^. The figure shows the specific grouping into five subclasses, which are indicated from subclass 1 to 5, already detected in the species-specific analysis of tomato Tau GSTs. Subclass 5 does not include GSTs from Arabidopsis.

In the work of Dixon and Edwards^34^, all Arabidopsis GSTs were assigned with a specific role. Considering these functional assignments, subclass 1 includes nine Arabidopsis GSTs (AT3G43800.1, AT1G78370.1, AT1G78340.1, AT1G78380.1, AT1G78320.1, AT1G78360.1, AT1G17180.1, AT1G17190.1 and AT1G53680.1) that are reported to be expressed under abiotic and biotic stresses, since they bind herbicides (AT1G17190.1), 1-chloro-2,4-dinitrobenzene (AT1G78380.1, AT1G17180.1, AT1G53680.1), and salicylic (AT3G43800.1) or jasmonic acid (AT1G78370.1).

Subclass 2 includes eight Arabidopsis GSTs (AT1G59700.1, AT1G59670.1, AT1G69930.1, AT1G69920.1, AT1G27130.1, AT1G27140.1, AT1G10370.1 and AT1G10360.1) all reported to have a low capability of binding glutathione. These GSTs result to be abundant in the nucleus and also bind RNA.

Arabidopsis Tau GSTs preferentially expressed in root (AT3G09270.1, AT2G29480.1, AT2G29470.1, AT2G29490.1, AT2G29460.1, AT2G29440.1, AT2G29450.1 and AT2G29420.1) when the concentration of auxin and/or abscisic acid increase are all located in the subclass 3. Finally, the three GSTs (AT1G74590.1, AT5G62480.1 and AT5G62480.2), which result to be highly expressed in seed under stress condition, are all included in subclass 4.

Subclass 5 includes S*. lycopersicum*, *V*. *vinifera* and *O*. *sativa* members while Arabidopsis GSTs are all absent. This aspect was further investigated also considering Tau GSTs from *B. oleracea*, another Brassicaceae in which 28 Tau GSTs were also characterized^35^. The phylogenetic tree, including Tau GSTs from *B. oleracea*, *V. vinifera*, *S. lycopersicum* and *A. thaliana* (Figure S2), shows that GSTs from *B. oleracea* are not included in the subclass 5, and suggests that the absence of members of subclass 5 could be a common feature in Brassicaceae.

47 GSTs are included in subclass 5 (Fig. 5). LOC_Os12g02960.1, from *O. sativa* ^36^, and Solyc01g081250.2.1 and Solyc09g063150.2.1, from *S. lycopersicum* ^37^ result to be expressed under abiotic stress. Moreover, six *V. vinifera* GSTs in the subclass were characterized as each one is able to bind and transport flavonoids in the berry’s skin (VIT_201s0026g01340.1, VIT_207s0005g04890.1, VIT_215s0024g01630.1, VIT_215s0024g01650.1 and VIT_215s0107g00150.1, in the work of Costantini^38^, and VIT_215s0024g01540.1 in the work of Malacarne^39^). Interestingly, four *V. vinifera* GSTs (VIT_205s0051g00240.1, VIT_207s0005g04880.1, VIT_205s0049g01090.1, VIT_205s0049g01120.1)^40^ and one *S. lycopersicum* GST (Solyc01g081270.2.1)^41^ result to be expressed during the abscission. This could suggest a functional divergence of members of subclass 5 and a possible association with abscission mechanisms thus explain its absence in Brassicaceae in contrast with their presence in grapevine and tomato^42^.

GST-Tau from *M. polymorpha* (*Marchantiophyta*) and *S. moellendorffii* (*Lycopodium*) are all grouped in subclass 1. This may suggest that this Tau subclass could be the group of ancestral GSTs sequences.

## Discussion

This analysis of 1107 GSTs from plants with sequenced genomes results in a wide phylogenetic tree providing insights on the organization of the different GST classes and highlights the presence of subclasses in the major classes currently described.

Beyond the assignment to specific GST classes for 666 unclassified proteins, the main aspect presented in this study is the possible confirmation of the paraphyletic origin of *Bryophytes* in contrast with the general assumption that *Bryophytes* and *Marchantiophytes* are a separated clade from the one that gave rise to higher plants. Moreover, the results indicate that *Marchantiophytes* could indeed belong to the branching bringing to higher plants.

The study includes the analysis of GST-Tau class, resulting in the discovery of the presence of at least 5 subclasses. The study tried to define the function of these subclasses. The results highlight the presence of a GST-Tau subclass including all the GST sequences from ancestor species, suggesting a primordial functionality for the members of this subclass. Finally a possible subclass, including genes associated with abscission, appears to be absent in Brassicaceae.

## Materials and Methods

### Genomic resources

GST protein sequences were searched by keyword. For *Amborella trichopoda* (v1.0), *Selaginella moellendorffii* (v1.0), *Sphagnum fallax* (v0.5), *Spirodela polyrhiza* (v2), *Zea mays* (Ensembl-18), *Micromonas pusilla* CCMP1545 (v3.0), *Marchantia polymorpha* (v3.1) and *Populus trichocarpa* (v3.0) the sequences were downloaded from Phytozome 11^43^ (https://phytozome.jgi.doe.gov/pz/portal.html); GSTs from *Picea abies* (v1.0) were downloaded from Congenie (http://congenie.org/); GSTs *Klebsormidium flaccidum* were downloaded from CGA (http://genome.microbedb.jp/Klebsormidium) while the ones from *Oryza sativa* were downloaded from TIGR^44^ (http://rice.plantbiology.msu.edu/); GST sequences from *Coffea canephora* were obtained searching in the Coffee genome Hub database^45^ (http://coffee-genome.org/coffeacanephora); *Glicine max*’s GSTs protein sequence were downloaded from Gramene^46^ (http://www.gramene.org/); GST sequences of *Solanum lycopersicum* (iTAG2.4) and *Capsicum annuum* (v1.55) were downloaded from SGN^47^ (https://solgenomics.net/), while the ones of *Solanum tuberosum* (PGSC_DM_v_3.4) were obtained from Spud db^48^ (http://solanaceae.plantbiology.msu.edu/); GST sequences of *Arabidopsis thaliana* were downloaded from TAIR10 (https://www.arabidopsis.org/). *Vitis vinifera* GST sequences (v2) were obtained from Cribi (http://genomes.cribi.unipd.it/grape/). GST sequences of *Physcomitrella patens* were obtained from^19^ and the ones from *Citrus sinensis* were obtained from^9^.

### Phylogenetic Analysis

Multiple alignments were obtained using Muscle^24^ with default parameter (gap open penalty -2,9, gap extension penalty 0). The Phylogenetic tree was built with RaxML^25^, using the maximum likelihood method, considering PROTCATBLOSUM62 as similarity matrix with the Bootstrap option. Finally the editing tool iTOL v3^49^ was used.

In order to obtain the pairwise distances of GST-Tau protein sequences we used “protdist” from PHYLIP, using the JTT matrix^50^. All the alignments, trees and matrices were built using shorter identifiers to indicate each gene. The conversion table between the original gene IDs and the code here used is reported in the supplemental Table 1.

### Class assignation for ambiguous cases

In order to understand the class of the three putative GST-Tau of the two algae and the class of the two putative Tau GSTs of the *Marchantiophyta* we performed a BLASTp^26^ with default parameters versus the entire GSTs collection here considered. A Uniprot BLASTp was also performed using default parameters versus UNIPROTkb^27^. The *M. pusilla* putative GST-Tau was further investigated by an InterProScan^28^ analysis with default parameters.

## Electronic supplementary material

Supplementary Figures

Supplementary Tables
