# Supplementary material for: Shaping the evolutionary tree of green plants: evidence from the GST family
Source: Sci Rep. 2017 Oct 30;7:14363. doi: 10.1038/s41598-017-14316-w (PMC5662610; doi:10.1038/s41598-017-14316-w)
Supplement: Supplementary file 1 — Supplementary Figures [file 41598_2017_14316_MOESM1_ESM.doc]

**Shaping the evolutionary tree of green plants: evidence from the GST family**

Authors:

Monticolo Francesco: Department of Agricultural Sciences, University of Naples “Federico II”, 80055, Portici (Na), Italy.

Colantuono Chiara: Department of Agricultural Sciences, University of Naples “Federico II”, 80055, Portici (Na), Italy.

Chiusano Maria Luisa*: Department of Agricultural Sciences, University of Naples “Federico II”, 80055, Portici (Na), Italy and Research Infrastructures for Marine Biological Resources (RIMAR), Stazione Zoologica Anton Dohrn, Villa Comunale, 80121, Naples, Italy.

*Corresponding author: Chiusano Maria Luisa, chiusano@unina.it

Supplementary Figure 1


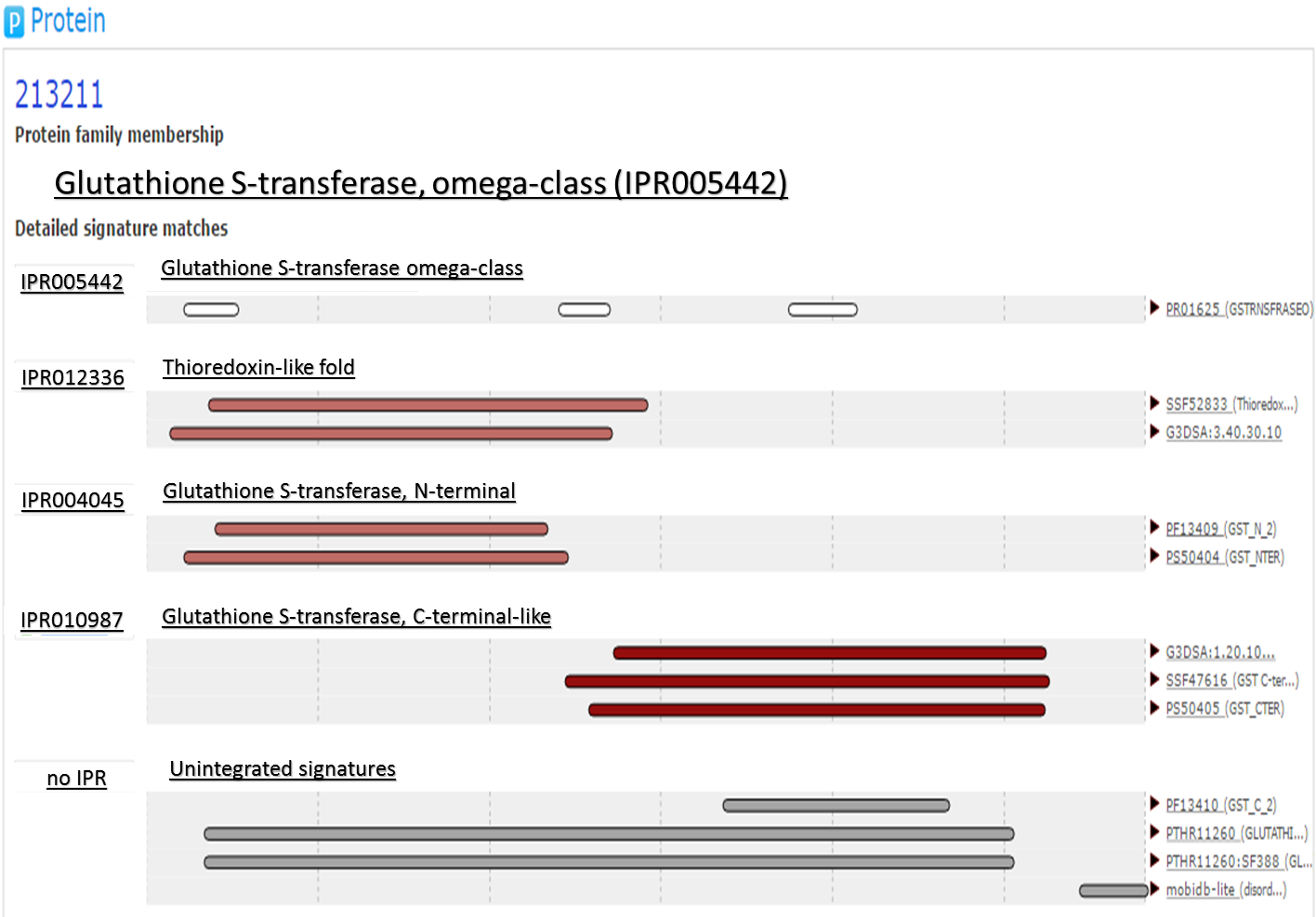


Supplementary Figure 1: InterProScan results of *Micromonas pusilla* (213211) GST sequence.

Supplementary Figure 2
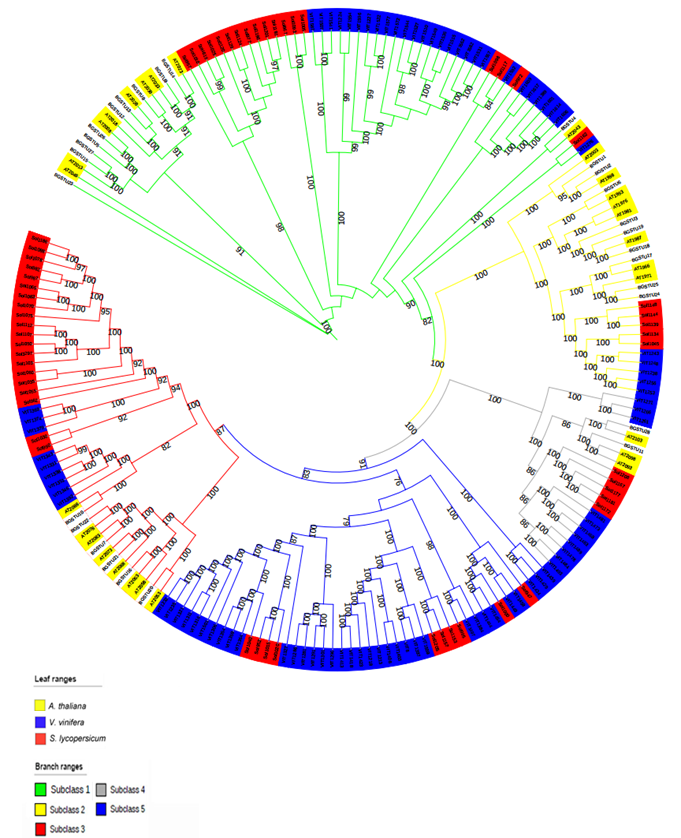


Supplementary Figure S2: Phylogenetic tree of GSTs from class Tau of *B. oleracea* (white), *A. thaliana* (yellow), *V. vinifera* (blue) and *S. lycopersicum* (red). The branches indicate the possible different subclasses, according to the color reported in the corresponding legend. Bootstrap values are also indicated.
